# Effects of Spray Drying and Freeze Drying on Physicochemical Properties, Antioxidant and ACE Inhibitory Activities of Bighead Carp (*Aristichthys nobilis*) Skin Hydrolysates

**DOI:** 10.3390/foods11142083

**Published:** 2022-07-13

**Authors:** Ye Dong, Wen Yan, Yi-Qi Zhang

**Affiliations:** Collaborative Innovation Center of Seafood Deep Processing, Key Laboratory of Aquatic Products Processing of Zhejiang Province, Institute of Seafood, Zhejiang Gongshang University, Hangzhou 310035, China; dy12686@163.com (Y.D.); ywseafood@126.com (W.Y.)

**Keywords:** fish skin hydrolysate, freeze drying, spray drying, antioxidant, ACE inhibitory activity

## Abstract

The physicochemical, structural properties, antioxidant, and angiotensin I-converting enzyme (ACE) inhibitory activities of fish skin protein hydrolysate (SPH) that were freeze-dried (SPH-FD) and spray-dried (SPH-SD) were investigated. SPH-SD showed abundant volatile compounds, higher DPPH radical scavenging activity and ferrous iron chelating activity than SPH-FD, while the ABTS radical scavenging activity and ACE inhibitory activity were not influenced by the drying method. Amino acid compositions showed a higher proportion of proline and hydroxyproline residues in SPH-FD. The major molecular weights were both distributed below 1000 Da. SPH-SD had spherical structures, while SPH-FD had glass shard-like structures. The results indicated that the drying method could affect the physicochemical properties of hydrolysates, and SPH-SD showed potential prospects in developing functional fortified foods.

## 1. Introduction

Hypertension is a major risk factor for cardiovascular disease, and reactive oxygen species (ROS) such as free radicals and peroxides can also cause cellular damage when they reach toxic levels, increasing risks for diseases [1]. The exploration of natural antioxidants or angiotensin converting enzyme (ACE) inhibitory molecules from processing by-products is gaining interest compared to traditional synthetic drugs that may have toxicological effects. There is some evidence that proteins may be an excellent source of antioxidants and antihypertensive peptides [2]. Due to mild process conditions and easy control of desired reactions, enzymatic hydrolysis is an efficient method to release bioactive peptides from protein molecules. Currently, several protein hydrolysates, including adzuki bean [3], hemp bran [4], rainbow trout viscera [5], fish sauce by-products [6], and sturgeon skin [7], have been reported to exhibit both ACE inhibition and antioxidant activity.

Inevitably, the moisture in protein hydrolysates after the enzymatic hydrolysis process needs to be removed by drying technology to improve the stability of the product and prolong its shelf life [8]. Spray drying and freeze drying are the most common methods of protein powder production. Freeze drying is an effective method for preserving the nutritional content of powder products. However, its industrial-scale application is hindered by high instrument costs and energy consumption [9]. Spray drying is a microcapsule technology widely used in the food industry. The main advantage of spray drying technology is the ability to control particle size based on shape and morphology, but high temperatures can destroy heat-sensitive substances [10]. Therefore, finding an effective and practical drying process to eliminate defects while maintaining biological activity has potential benefits.

Bighead carp (*Aristichthys nobilis*) is one of the popular freshwater species grown in China. During fish filleting and bighead carp head processing, underutilized fish by-products include the head, skin, frames, trimmings, and fins, which account for more than 60% of total biomass [11]. These fish by-products, especially fish skins, are a good source of collagen or gelatin and are widely used in the food, cosmetic, and biomedical industries. They are also the raw materials for the production of bioactive peptides or protein hydrolysates, which can be used as functional food ingredients [12,13]. Although processing protein hydrolysates into powders is a common procedure, to our knowledge, limited research has been undertaken on developing drying strategies to balance the preparation process, functionality, and bioactivity of hydrolysates [14,15].

The aim of this study was to compare the effect of freeze drying and spray drying on the structural properties, antioxidant and ACE inhibitory activities of protein hydrolysates from bighead carp skin. The findings may help to provide better drying strategies for the application of fish skin hydrolysates in the food industry.

## 2. Materials and Methods

### 2.1. Materials

Bighead carp was purchased from a local aquatic market in Hangzhou, Zhejiang, China. After the fish were stunned and sampled, the skins were collected manually, treated with 0.1 M NaOH for 4 h to remove non-collagen proteins, and washed with running water to neutral pH. The alkali-treated skins were treated with 10% isopropanol for 12 h to degrease, washed with running water to remove the residual isopropanol, then dried at 40 °C, crushed, and stored in a dryer. Protamex was purchased from Novozyme (Tianjin, China). 1,1-Diphenyl-2picrylhydrazy (DPPH) and 2,2′-azinobis-(3-ethylbenzothiazoline-6-sulfonic acid) diammonium salt (ABTS) were obtained from Aladdin Reagents Co., Ltd. (Shanghai, China). Hippuryl-histidylleucine (HHL), aprotinin, bacitracin, and cytochrome C, were obtained from Sigma-Aldrich (Milwaukee, WI, USA). Acetonitrile (ACN) and trifluoroacetic acid (TFA) were of chromatographical grade. All the other chemicals were of analytical grade and were purchased from Sinopharm Chemical Reagent Co., Ltd. (Shanghai, China).

### 2.2. Preparation of Skin Protein Hydrolysates (SPH)

Bighead carp skins were placed in distilled water (2%, *w*/*v*) and hydrolyzed by Protamex (pH 7.0) at 50 °C for 4 h with an enzyme–substrate ratio of 2% (*w*/*w*). The pH was kept constant by adding 0.1 M NaOH. The degree of hydrolysis (DH) of SPH was analyzed according to pH-stat method. The protein content (% total nitrogen × 5.79) of bighead carp skin was 74.23% ± 1.21%, determined by the AOAC method (AOAC, 2000). After 4 h of reaction, the enzyme was immediately inactivated by heating at 95 °C for 15 min, followed by centrifugation at 8000× *g* for 15 min (DH was 11%). The supernatant was collected and stored at −30 °C for further analysis.

### 2.3. Drying Process

Freeze drying was undertaken using a freeze dryer (Labconco Free Zone 6 L, Kansas City, MO, USA). SPH samples (500 mL) were placed in 3.5 cm × 9 cm × 17 cm disposable preserving boxes and frozen at −30 °C for 24 h. The frozen samples were placed on shelves in the freeze drier and lyophilized at −80 °C for 72 h with heating plate temperature of 20 °C. Spray drying was performed using a spray dryer (BUCHI B-290, Labortechnik AG, Flawil, Switzerland) with an inlet temperature of 180 °C, an output temperature of 100 °C, and the flow rate of 40%. The freeze-dried and spray-dried SPH were placed in sealed bags and stored at −20 °C until analysis. The samples were labeled SPH-FD and SPH-SD, respectively.

### 2.4. Color Analysis

The color of SPH was determined using Hunterlab (ColorQuest XE, Reston, VA, USA) in terms of lightness (L*), redness (a*), and yellowness (b*) values. Whiteness index was measured using the following calculation:

Whiteness Index = 100 − [(100 − L*)^2^ + a*^2^ + b*^2^]^1/2^ (1)

### 2.5. Determination of Amino Acid Composition

The amino acid composition of SPH-FD and SPH-SD was determined using an Agilent 1100 series HPLC system (Agilent Technologies, Inc., Santa Clara, CA, USA) equipped with a C18 ODS Hypersil column (4.6 mm × 250 mm, 5 µm particle size; Agilent Technologies, Inc.). The hydrolysates were hydrolyzed in sealed tubes with 6 M HCl at 110 °C for 22 h under vacuum. Amino acid analysis was performed by pre-column derivatization with o-phthalaldehyde and fluorobenzyl chloroform. Hydroxyproline was detected at 262 nm and other amino acids at 338 nm.

### 2.6. Scanning Electron Microscopy (SEM)

The SPH-FD and SPH-SD were fixed to the aluminum sample stubs, and then coated with gold. Then, the surface morphology was observed by using an SEM (FEI Quanta 650, Hillsboro, OR, USA) under the conditions of accelerated voltage of 2000 kV and magnification of 2000×.

### 2.7. Fourier Transform Infrared Spectroscopy (FTIR)

The FTIR spectra of SPH-FD and SPH-SD were analyzed using FTIR spectroscopy. The sample was mixed with dry potassium bromide, pressed into thin sections, and determined according to Elavarasan et al. [16]. The spectral range is 4000 to 400 cm^−1^ with the average of 32 automated scans at a resolution of 4 cm^−1^. Spectra were baseline corrected and normalized to the amide I band using OMNIC software version 8.2 (Thermal Scientific, Madison, WI, USA).

### 2.8. Molecular Weight Distribution

The molecular weight (MW) distribution of SPH-FD and SPH-SD was determination by size exclusion chromatography using a Waters e2695 HPLC system equipped with a TSK-gel G2000 SW_XL_ column (7.8 mm × 300 mm, Tosoh, Tokyo, Japan). The chromatographic conditions were as follows: the eluent was 45% acetonitrile/55% water/0.1% trifluoroacetic acid, the flow rate was 0.5 mL/min, and the UV detector was monitored at 220 nm. HHL (429 Da), bacitracin (1422 Da), aprotinin (6.5 kDa), and cytochrome C (12.4 kDa) were used as standards to obtain the MW calibration curve: lg (M) = −0.2008t + 6.8668, *R*^2^ = 0.9761.

### 2.9. Volatile Compounds Analysis

The volatile compounds of the samples were detected by headspace solid-phase microextraction-gas chromatography mass spectrometry (HS-SPME-GC-MS). Briefly, 4 mL of samples were placed into a 15 mL glass vial, and then the sealed vial was preserved at 50 °C for 10 min. The SPME device with polydimethylsiloxane (PDMS) was used to expose the fiber in the upper space of the vial at 70 °C to absorb the sample. After sampling, the fiber was placed in the GC injector, and GC-MS analysis was carried out using a TR-35 MS (30 m × 0.25 mm, 0.25 μm film, Agilent Technologies, Santa Clara, CA, USA) column and He as the carrier at 1 mL/min. The temperature program was set as follows: the inlet temperature was set to 250 °C with an initial temperature of 35 °C and held for 3 min, rising to 70 °C at a rate of 2.5 °C/min, then increased to 150 °C at a rate of 8 °C/min. Finally, the temperature was increased by a ramp of 20 °C/min to 230 °C and held for 5 min. The mass spectra in electron impact mode at 70 eV in the mass range of 45–550 m/z at a scan rate of 0.22 s/scan. The transmission line temperature and ion trap temperature of GC-MS were set to 250 and 150 °C, respectively.

### 2.10. Antioxidant Activity

#### 2.10.1. DPPH Radical Scavenging Activity

The DPPH radical scavenging activity of SPH was determined according to Saisavoey, et al. [17] with some modifications. Briefly, 1.0 mL SPH was added to 1.0 mL of DPPH solution. After mixing, the mixture was then left in the dark for 30 min at room temperature and the absorbance was measured at 517 nm. The scavenging effect for DPPH radical was expressed as follows:

DPPH radical scavenging activity (%) = [1 − (A_1_ − A_2_)/A_0_] × 100 (2)

where A_1_: the absorbance of SPH at 517 nm; A_2_: the absorbance of SPH blank without DPPH; A_0_: the absorbance of the control without SPH.

#### 2.10.2. ABTS Radical Scavenging Activity

The ABTS radical scavenging activity of the SPH was measured according to the method of Zhang, Dong and Dai [12]. Briefly, 50 μL of SPH was added to 150 μL of diluted ABTS solution (absorbance of 0.70 ± 0.02 at 734 nm). After blending, the mixture was incubated for 6 min and then the absorbance was measured at 734 nm. The scavenging effect on ABTS radical was expressed as follows:

ABTS radical scavenging activity (%) = [1 − (A_1_ − A_2_)/A_0_] × 100 (3)

where A_1_: the absorbance of SPH at 734 nm; A_2_: the absorbance of SPH blank without ABTS; A_0_: the absorbance of the control without SPH.

#### 2.10.3. Ferrous Iron’s Chelating Activity

The ferrous iron’s chelating activity of SPH was determined according to Zhang, Dong and Dai [12]. Briefly, 1 mL of SPH was mixed with 0.1 mL 2 mM FeCl_2_ and 3.7 mL deionized water. The mixture was then reacted with 0.2 mL of 5 mM ferrozine and kept for 20 min at room temperature and monitored at 562 nm. The control group was prepared by replacing the SPH with deionized water. The metal chelating activity was calculated as follows:

Metal chelating activity (%) = (A_0_ − A_1_)/A_0_ × 100 (4)

where A_1_: the absorbance of SPH at 562 nm; A_0_: the absorbance of SPH control without SPH.

### 2.11. ACE Inhibitory Activity

The ACE inhibitory activity of SPH was measured according to Zhang et al. [18]. Briefly, 25 μL ACE solution (100 U/L) was mixed with 40 μL SPH sample, incubated in a 37 °C water bath for 10 min, and 40 μL of 6.5 mM HHL was added to the mixture to initiate the reaction. After 30 min of incubation, the reaction was terminated by the addition of 85 μL of 1 M HCl. Hippuric acid (HA) was separated using a Waters e2695 HPLC system equipped with a SunFire C18 column (4.6 mm × 250 mm, 5 μm). The eluent was 30% acetonitrile/70% water/0.1% trifluoroacetic acid, the flow rate was 0.8 mL/min, and the detection wavelength was 228 nm. ACE inhibitory activity (%) was calculated as follows:

ACE inhibitory activity (%) = (A_1_ − A_2_)/A_1_ × 100 (5)

where A_1_: the HA content of the control; A_2_: the HA content of the reaction with the SPH.

### 2.12. Statistical Analysis

Data were presented as mean ± SD. Statistical analysis was performed using ANOVA with Duncan’s multiple range tests using SPSS software version 21.0 (SPSS Inc., Chicago, IL, USA). All analyses were performed in triplicate. The statistical significance was set at a level of *p* < 0.05.

## 3. Results and Discussion

### 3.1. Physicochemical Properties

The color of protein hydrolysate would affect the acceptability of the product. SPH-SD had higher redness (a*) and yellowness (b*) values, while SPH-FD had higher lightness (L*) (Table 1). It indicated that the color of the hydrolysates was positively influenced by drying method. The color difference is mainly due to the Maillard reaction that occurs in the spray drying process [19]. In contrast, the higher lightness of SPH-FD is thought to be due to the low temperature and vacuum during freeze drying [14,20]. The lightness value of SPH-FD was similar to that of freeze-dried chicken breast protein hydrolysate [21]. Elavarasan, Shamasundar, Badii and Howell [16] also reported that the color of freeze-dried *Cirrhinus mrigala* protein hydrolysate showed higher L* and lower a* and b* values compared to oven drying.

As shown in Table 1, the protein content of SPH-FD and SPH-SD was not significantly different. The moisture content of FD is higher than that of SD. The air temperature in SD was higher than that in the FD process, which could more effectively remove the moisture in the SPH. In addition, the sublimation of ice crystals forms a porous structure during FD, and the higher cooling rate and increased nucleation make the outer pore size of the sample smaller, which in turn hinders the sublimation of ice crystals [22]. Therefore, the moisture content of SPH-FD is higher than that of SPH-SD. It was consistent with the result that the moisture content of freeze-dried chicken hydrolysate was higher than that of spray-dried [21]. In contrast, Kleekayai et al. [23] observed that the moisture content of spray-dried whey protein hydrolysate was higher than that of freeze-dried whey protein hydrolysate. This may be related to the spray drying parameters employed, such as inlet and outlet temperatures, feed concentration, and airflow rate, which may affect the moisture content of SD powders [24].

### 3.2. Amino Acid Compositions of SPH-SD and SPH-FD

The amino acid composition of SPH dried by SD and FD are shown in Table 2. The total amino acid contents of SPH-SD and SPH-FD were 89.33 and 90.40 g/100 g, respectively, of which the essential amino acids accounted for 18.36% and 16.24%, respectively. SPH was rich in glycine (298.18–311.91 residues), alanine (116.09–119.99 residues), proline (115.15–142.66 residues), glutamic (80.18–83.06 residues), and hydroxyproline (75.49–91.32 residues), but poor in cysteine (0.52–0.58 residues) and tyrosine (3.27–4.27 residues). Glycine is the most abundant amino acid in SPH-SD (311.91 residues) and SPH-FD (298.18 residues), accounting for about one-third of the total residues. The amino acid composition was in line with the characteristics of collagen, i.e., glycine accounts for one-third of the total amino acid and lacks cystine and tyrosine. Imino acids (proline and hydroxyproline) accounted for approximately 20–26% of the total amino acids in SPH. The extraction and drying process of collagen is usually carried out at low temperatures, which contributes to the thermal stability of collagen [25]. The imino acid content of SPH-FD (233.98 residues) was 1.23 times that of SPH-SD (190.64 residues), indicating that some imino acids were lost due to high temperature during the SD process.

The content of hydrophobic amino acids in two samples was relatively high, ranging from 445.65 to 457.53 residues. Studies have found that the antioxidant activity of hydrolysates mainly depends on the composition and sequence of amino acids [17]. Glycine and hydrophobic amino acids are important for the bioactivity of hydrolysates, especially their potential antioxidant activity [26]. In addition, acidic amino acids such as glutamic acid and aspartic acid can eliminate free radicals and quench unpaired electrons by donating protons [27]. In the present study, SPH was rich in acidic amino acids, glycine and hydrophobic amino acids, suggesting that it may have good antioxidant activity.

### 3.3. SEM

**Figure 1:**
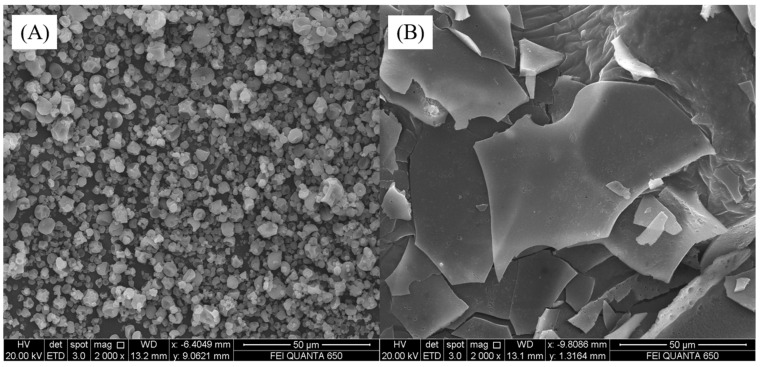
SEM image of SPH-SD (**A**) and SPH-FD (**B**).

The microstructure of fish skin protein hydrolysate powder obtained by freeze drying and spray drying is shown in Figure 1. The SEM image of the powder reflects the influence of drying method on the morphological structure of the produced particles [28]. The morphology of the sample is one of the most important factors affecting its function and stability. It can be seen that the particle size of SPH-SD is smaller than that of SPH-FD, corresponding to the higher solubility of SPH-SD, and the morphological structure of SPH-SD is irregular and has smooth spherical particles. The higher inlet temperature and the rapid evaporation of water during the SD process forms a protective film on the surface of the sample, resulting in a concave structure and folds on the surface of smooth spherical particles. FD samples were fractured sheet/laminar structures without the formation of microspheres. Changes in drying conditions, such as inlet air temperature for spray drying, feed flow rate, etc., may affect the hygroscopicity and particle morphology of the sample, which in turn affects the physicochemical properties of the sample [29]. Therefore, the changes in the physicochemical properties, structure, and biological properties of fish skin protein hydrolysates under different spray drying conditions can be further studied in the follow-up research.

### 3.4. FTIR Analysis

**Figure 2:**
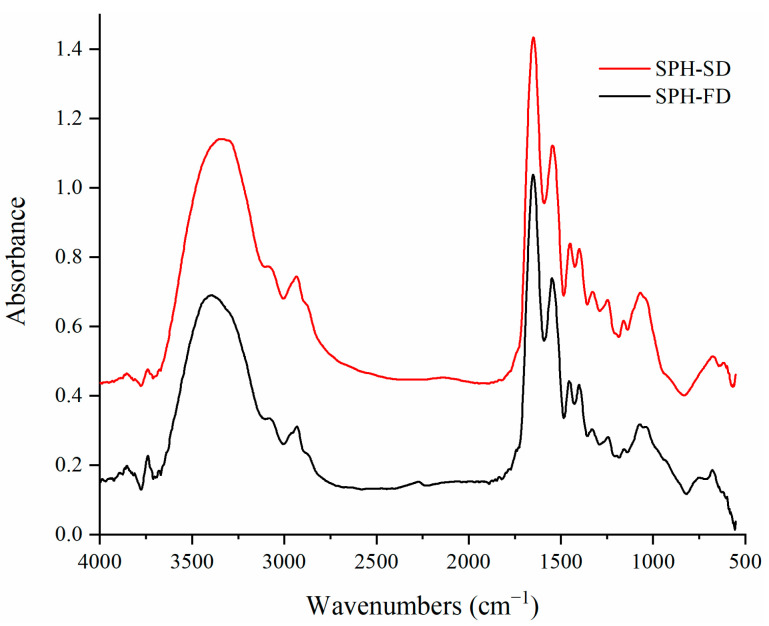
FTIR spectra of SPH-SD and SPH-FD.

FTIR spectroscopy can be used to characterize changes in secondary structure and functional groups of proteins. As shown in Figure 2, the hydrolysates of the two samples showed similar patterns in FTIR spectra, indicating similar chemical compositions. The 3340–3410 cm^−1^ shows the amide A bands, indicating N-H and O-H stretching through hydrogen bonding. When the N-H group of the hydrolysate participates in hydrogen bonding, the position of the band will shift to a lower frequency. Compared with SPH-FD (3409.67 cm^−1^), SPH-SD (3340.24 cm^−1^) has a lower wavenumber, and it is more likely that N-H group short peptides are involved in hydrogen bonding. The 2933.32 and 2931.39 cm^−1^ show amide B bands, which are associated with asymmetric stretching of = C-H and NH_3_^+^ of SPH-SD and SPH-FD, respectively. The 1648.91 and 1652.77 cm^−1^ show amide I, caused by C=O stretching vibration or hydrogen bonding. The peak position of amide I shifted from 1648.91 (SPH-SD) to 1652.77 cm^−1^ (SPH-FD). The increase in wavenumber revealed that part of the β-sheet in SPH turned into α-helix or random coil [9]. The 1546.70–1548.62 cm^−1^ shows amide II bands, corresponding to N-H vibrations. The 1200 to 1400 cm^−1^ shows amide III bands, which are associated with C-N stretching, N-H and O=C-N bending of the amide bond. The intensity of the amide III band in SPH-SD was greater than that in SPH-FD, indicating that the intermolecular interactions of peptides in SPH-SD were stronger, probably caused by the high temperature of SD. The 1180–953 cm^−1^ shows the “saccharide” bands [16], indicating the presence of aldehyde groups in the sample, probably formed by lipid oxidation during hydrolysis and drying [30], SPH-SD shows two bands at 1160.99 and 1070.34 cm^−1^, while SPH-FD has only one band at 1076.13 cm^−1^. In SPH-SD, the saccharide peak was very strong, while in SPH-FD it was weaker. This may be due to the higher temperature (180 °C) and the higher degree of lipid oxidation in the SD process compared to FD.

### 3.5. Molecular Weight Distribution in SPH-FD and SPH-SD

**Figure 3:**
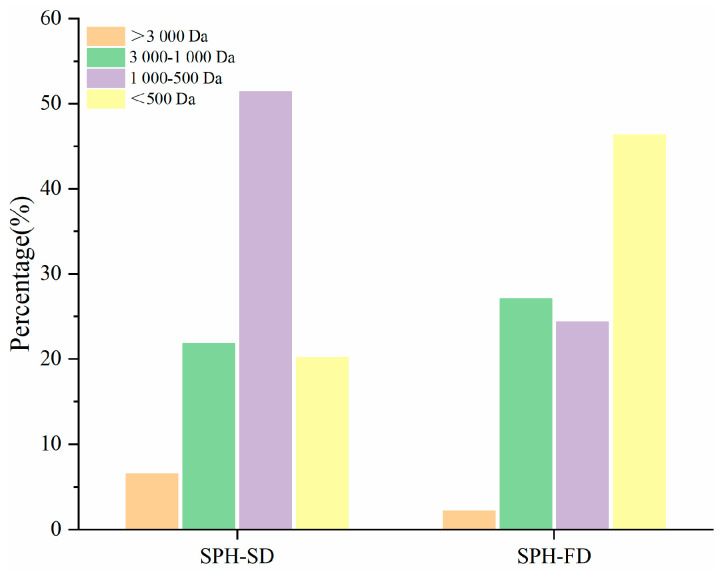
Distribution of MW of SPH-SD and SPH-FD.

Figure 3 shows the relative molecular weight of SPH. The molecular weight distributions of SPH-FD and SPH-SD were slightly different. The fractions greater than 3000 Da and less than 500 Da in SPH-FD accounted for about 2.18% and 46.36%, respectively, while the corresponding fractions in SPH-SD accounted for 6.56% and 20.18%, respectively. The component content of SPH-SD in the range of 500–1000 Da was 51.43%, which was about 2.11 times higher than that of SPH-FD. There was no significant difference in molecular weight between the two groups at 1000–3000 Da. The molecular weight of SPH-SD is slightly larger than that of SPH-FD, which was mainly caused by the aggregation and cross-linking of peptides during drying [31].

### 3.6. Volatile Compounds

**Figure 4:**
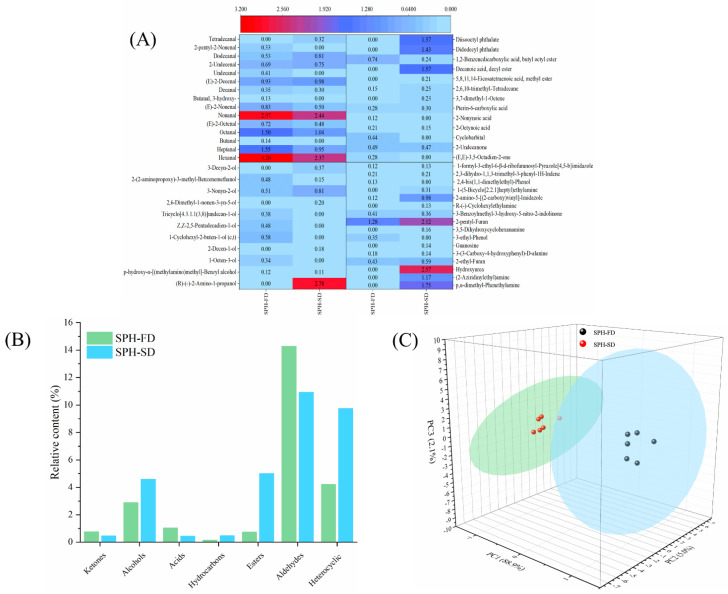
Heatmap (**A**), contents of various classes of volatile substances (**B**), and PCA analysis (**C**) of volatile compounds of SPH-SD and SPH-FD.

The volatile flavor substances of SPH-FD and SPH-SD were determined by GC-MS. A total of 55 volatile compounds were identified. They were 15 aldehydes, 2 ketones, 11 alcohols, 5 esters, 4 acids, 2 hydrocarbons, 2 phenols, and 12 heterocyclic compounds (Figure 4A). Among them, 38 volatile compounds were detected in SPH-FD and 42 in SPH-SD. In total, 17 volatile compounds were only detected in the SPH-SD, whereas 13 volatile compounds were only found in the SPH-FD. Aldehydes and heterocyclic compounds are one of the main volatile compounds. A total of 16 aldehydes were detected in SPH-FD (14.28%) and 11 aldehydes were detected in SPH-SD (10.94%). The content of volatile components of aldehydes in SPH-FD was higher than that in SPH-SD. During the SD process, the amount of aldehyde in SPH-SD was reduced due to the Maillard reaction and other reactions [32]. Aldehydes mainly come from the Strecker degradation of amino acids or the oxidation of fat. Aldehydes with 6–9 carbon atoms have a fruit flavor, fragrance, and fat flavor. Compared with heterocyclic compounds, ketones, aldehydes, alcohols, and other volatile compounds contribute less to the overall aroma. The relative content of esters and heterocyclic compounds in SPH-SD samples was higher than that in SPH-FD, which may be caused by the thermal decomposition of amino acids during the SD process. The threshold of heterocyclic compounds is generally low, and they make a great contribution to the overall sensory flavor of SPH. For example, 2-pentylfuran has a flavor similar to ham, which can play a certain role in enhancing the flavor and can also improve antioxidant activity by double bond addition. *Perinereis aibuhitensis* hydrolysates had more volatile substances and higher antioxidant activity by spray drying [14]. SFH-SD has a higher content of volatile compounds than SPH-FD, especially heterocyclic compounds, which may have higher antioxidant activity.

In order to visually analyze the differences in flavor substance of SPH, principal component analysis (PCA) was further used to analyze the GC/MS data of the SPH-FD and SPH-SD groups. As shown in Figure 4C, the cumulative variance contribution of PC1 (88.9%), PC2 (5.0%), and PC3 (2.1%) was 96.0%, indicating that the groups of the SPH-FD and SPH-SD samples were well separated, and there were certain differences in the characteristic flavors of the samples. The SPH-SD group was clustered on the left side of the PCA plot, and the SPH-FD group was clustered on the right side of the PCA plot. These data indicate that the volatile compound profiles of SPH-FD and SPH-SD were different.

### 3.7. Antioxidant and ACE Inhibitory Activities

**Figure 5:**
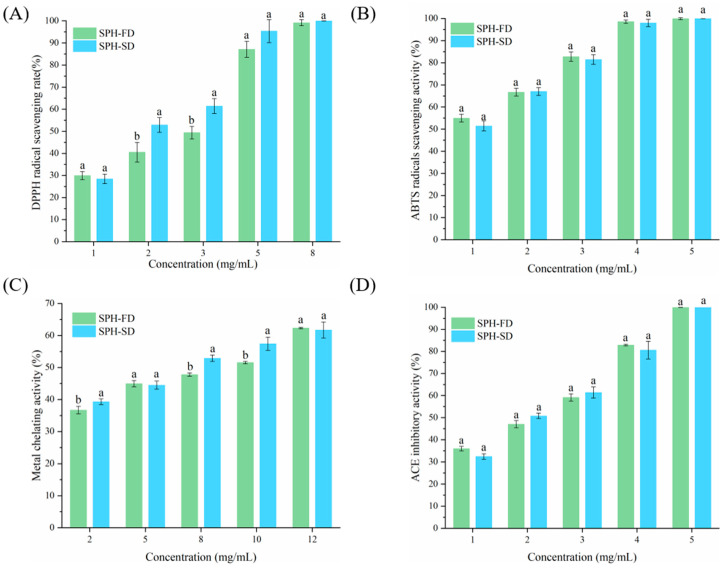
DPPH radical scavenging activity (**A**), ABTS radical scavenging activity (**B**), metal chelating activity (**C**), and ACE inhibitory activity (**D**) of SPH-SD and SPH-FD. Different letters denote that the results of SPH-FD and SPH-SD differ significantly (*p* < 0.05).

The fish skin hydrolysates under different drying conditions exhibited good potential antioxidant activities. Figure 5 showed the DPPH, ABTS radical scavenging, and ferrous iron’s chelating activities of SPH-FD and SPH-SD at different concentrations, respectively, and the scavenging activities showed concentration-dependent activity. This result was similar to that of Alinejad et al. [33]. The drying process affects the antioxidant capacity of the SPH. Overall, the DPPH radical scavenging activity and ferrous iron’s chelating activity of SPH-SD were significantly (*p* < 0.05) higher than those of SPH-FD, while SPH-SD and SPH-SD had no significant difference in the scavenging ability of ABTS. The IC_50_ values of SPH-FD for DPPH, ABTS radical scavenging, and ferrous iron’s chelating activities were 2.14, 1.05, and 7.03 mg/mL, and the corresponding values of SPH-SD were 1.81, 1.11, and 5.79 mg/mL, respectively. Liu, Wang, Yu, Li, Ji, Sun, Jiang, Li and Liu [14] and Alinejad, Motamedzadegan, Rezaei and Regenstein [33] reported similar results that spray-dried samples had higher DPPH scavenging activity than freeze-dried samples. Chen et al. [34] reported that the antioxidant activity of dipeptides was higher than that of the constituent amino acid mixture. The free amino acids in the hydrolysates were reorganized into dipeptides and tripeptides during SD, which would improve the antioxidant properties of the samples [14]. On the other hand, the Maillard reaction that occurs during the SD process may also increase the antioxidant activity of the samples [35]. These results suggest that the drying method affects the antioxidant activity of the hydrolysates. 

The ACE inhibitory activities of SPH-FD and SPH-SD samples at different concentrations (1.0–5.0 mg/mL) are shown in Figure 5D. When the concentration was 5.0 mg/mL, the ACE inhibitory activities of both SFH-FD and SFH-SD reached 100%. The IC_50_ values for ACE inhibitory activity in SFH-FD and SFH-SD were 1.79 and 1.75 mg/mL, respectively. There was no significant (*p* > 0.05) difference in ACE inhibitory activity between SPH-FD and SPH-SD. These results suggested that the drying method did not affect the ACE inhibitory activity of the hydrolysates, which was consistent with Elavarasan’s study [16]. Previous studies have demonstrated that the smaller peptides are thermally stable and the heat generated during the drying process has no significant effect on ACE inhibitory activity [16,36]. In this study, the small peptides with SPH-SD and SPH-FD MW less than 1000 Da reached more than 70%, which may have good thermal stability.

## 4. Conclusions

Fish skin proteins were hydrolyzed by Protamex and dried by spray drying and freeze drying. SPH-SD had a high content of volatile compounds and low lightness. FTIR analysis showed that the structures of the hydrolysates of the two drying methods changed slightly, and the antioxidant activity of SPH-SD was better, but the ACE inhibitory activity was not affected by the drying method. Amino acid compositions showed a higher proportion of proline and hydroxyproline residues in SPH-FD. The results indicated that the drying method could affect the physicochemical properties of hydrolysates, and SPH-SD showed potential prospects in developing functional fortified foods. This study provides a drying strategy for protein hydrolysates from aquatic product processing by-products. The structure–activity relationship and amino acid sequence of the hydrolysate need to be further studied.

## Figures and Tables

**Table 1:** Physicochemical properties of freeze-dried and spray-dried fish skin hydrolysate powder.

Physicochemical Properties	Drying Method	
	Freeze Drying	Spray Drying
Color Parameters		
L*	91.03 ± 0.29 a	87.49 ± 1.12 b
a*	0.34 ± 0.01 b	0.76 ± 0.04 a
b*	8.02 ± 0.04 b	9.62 ± 0.03 a
Moisture (%)	2.57 ± 0.53 a	1.59 ± 0.31 b
Protein content (%)	92.75 ± 0.21 a	93.12 ± 0.18 a

Different letters denote the significant difference (*p* < 0.05).

**Table 2:** Amino acid composition of SPH-SD and SPH-FD samples (results are expressed as residues/1000 total residues).

Amino Acids	SPH-SD	SPH-FD
Aspartic acid/asparagine	53.56	51.73
Glutamic acid/glutamine	83.06	80.18
Serine	30.31	28.87
Histidine	4.69	3.68
Glycine	311.91	298.18
Threonine	28.24	26.29
Arginine	55.75	53.33
Alanine	119.99	116.09
Tyrosine	4.27	3.27
Cysteine	0.52	0.58
Valine	23.57	22.82
Methionine	15.89	10.24
Phenylalanine	15.22	14.68
Isoleucine	12.86	12.52
Leucine	22.96	22.44
Lysine	26.56	21.13
Proline	115.15	142.66
Hydroxyproline	75.49	91.32
Total	1000	1000
Total essential amino acids	150.00	133.80
Imino acids	190.64	233.98

## Data Availability

The data are available from the corresponding author.

## References

[1] Hao L., Gao X., Zhou T., Cao J., Sun Y., Dang Y., Pan D. (2020). Angiotensin I-Converting Enzyme (ACE) Inhibitory and Antioxidant Activity of Umami Peptides after In Vitro Gastrointestinal Digestion. J. Agric. Food Chem..

[2] Mirzaei M., Mirdamadi S., Ehsani M.R., Aminlari M., Hosseini E. (2015). Purification and identification of antioxidant and ACE-inhibitory peptide from *Saccharomyces cerevisiae* protein hydrolysate. J. Funct. Foods.

[3] Cao M., Li W., Li H., Zhang J., Liu Y., Liu X. (2022). Antioxidant and ACE inhibitory activities of peptides prepared from adzuki bean by semi-solid enzymatic hydrolysis. Food Biosci..

[4] Samaei S.P., Martini S., Tagliazucchi D., Gianotti A., Babini E. (2021). Antioxidant and Angiotensin I-Converting Enzyme (ACE) Inhibitory Peptides Obtained from Alcalase Protein Hydrolysate Fractions of Hemp (*Cannabis sativa* L.) Bran. J. Agric. Food Chem..

[5] Vasquez P., Zapata J.E., Chamorro V.C., Garcia Filleria S.F., Tironi V.A. (2022). Antioxidant and angiotensin I-converting enzyme (ACE) inhibitory peptides of rainbow trout (*Oncorhynchus mykiss*) viscera hydrolysates subjected to simulated gastrointestinal digestion and intestinal absorption. LWT.

[6] Khositanon P., Panya N., Roytrakul S., Krobthong S., Chanroj S., Choksawangkarn W. (2021). Effects of fermentation periods on antioxidant and angiotensin I-converting enzyme inhibitory activities of peptides from fish sauce by-products. LWT.

[7] Gui M., Gao L., Rao L., Li P., Zhang Y., Han J.-W., Li J. (2022). Bioactive peptides identified from enzymatic hydrolysates of sturgeon skin. J. Sci. Food Agric..

[8] Gan J.Y., Chang L.S., Mat Nasir N.A., Babji A.S., Lim S.J. (2020). Evaluation of physicochemical properties, amino acid profile and bioactivities of edible Bird’s nest hydrolysate as affected by drying methods. LWT.

[9] Liu L., Dai X., Kang H., Xu Y., Hao W. (2020). Structural and functional properties of hydrolyzed/glycosylated ovalbumin under spray drying and microwave freeze drying. Food Sci. Human Wellness.

[10] Özdemir E.E., Görgüç A., Gençdağ E., Yılmaz F.M. (2022). Physicochemical, functional and emulsifying properties of plant protein powder from industrial sesame processing waste as affected by spray and freeze drying. LWT.

[11] Dong Y., Yan W., Zhang X.-D., Dai Z.-Y., Zhang Y.-Q. (2021). Steam Explosion-Assisted Extraction of Protein from Fish Backbones and Effect of Enzymatic Hydrolysis on the Extracts. Foods.

[12] Zhang Y., Dong Y., Dai Z. (2021). Antioxidant and Cryoprotective Effects of Bone Hydrolysates from Bighead Carp (*Aristichthys nobilis*) in Freeze-Thawed Fish Fillets. Foods.

[13] Rossi Y.E., Vanden Braber N.L., Díaz Vergara L.I., Montenegro M.A. (2021). Bioactive Ingredients Obtained from Agro-industrial Byproducts: Recent Advances and Innovation in Micro- and Nanoencapsulation. J. Agric. Food Chem..

[14] Liu T., Wang Y., Yu X., Li H., Ji L., Sun Y., Jiang X., Li X., Liu H. (2022). Effects of freeze-drying and spray-drying on the physical and chemical properties of *Perinereis aibuhitensis* hydrolysates: Sensory characteristics and antioxidant activities. Food Chem..

[15] Wang Y., Selomulya C. (2020). Spray drying strategy for encapsulation of bioactive peptide powders for food applications. Adv. Powder Technol..

[16] Elavarasan K., Shamasundar B.A., Badii F., Howell N. (2016). Angiotensin I-converting enzyme (ACE) inhibitory activity and structural properties of oven- and freeze-dried protein hydrolysate from fresh water fish (*Cirrhinus mrigala*). Food Chem..

[17] Saisavoey T., Sangtanoo P., Reamtong O., Karnchanatat A. (2019). Free radical scavenging and anti-inflammatory potential of a protein hydrolysate derived from salmon bones on RAW 264.7 macrophage cells. J. Sci. Food Agric..

[18] Zhang Y., Tu D., Shen Q., Dai Z. (2019). Fish Scale Valorization by Hydrothermal Pretreatment Followed by Enzymatic Hydrolysis for Gelatin Hydrolysate Production. Molecules.

[19] Rodríguez-Díaz J.C., Tonon R.V., Hubinger M.D. (2014). Spray Drying of Blue Shark Skin Protein Hydrolysate: Physical, Morphological, and Antioxidant Properties. Drying Technol..

[20] Li L., Zhang M., Bhandari B. (2019). Influence of drying methods on some physicochemical, functional and pasting properties of Chinese yam flour. LWT.

[21] Setthaya P., Jaturasitha S., Ketnawa S., Chaiyaso T., Sato K., Wongpoomchai R. (2021). Influence of Commercial Protease and Drying Process on Antioxidant and Physicochemical Properties of Chicken Breast Protein Hydrolysates. Foods.

[22] Ezhilarasi P.N., Indrani D., Jena B.S., Anandharamakrishnan C. (2013). Freeze drying technique for microencapsulation of Garcinia fruit extract and its effect on bread quality. J. Food Eng..

[23] Kleekayai T., O’Neill A., Clarke S., Holmes N., O’Sullivan B., FitzGerald R.J. (2022). Contribution of Hydrolysis and Drying Conditions to Whey Protein Hydrolysate Characteristics and In Vitro Antioxidative Properties. Antioxidants.

[24] Rezvankhah A., Emam-Djomeh Z., Askari G. (2020). Encapsulation and delivery of bioactive compounds using spray and freeze-drying techniques: A review. Dry. Technol..

[25] Liu D., Liang L., Regenstein J.M., Zhou P. (2012). Extraction and characterisation of pepsin-solubilised collagen from fins, scales, skins, bones and swim bladders of bighead carp (*Hypophthalmichthys nobilis*). Food Chem..

[26] Mirzapour-Kouhdasht A., Moosavi-Nasab M., Kim Y.-M., Eun J.-B. (2021). Antioxidant mechanism, antibacterial activity, and functional characterization of peptide fractions obtained from barred mackerel gelatin with a focus on application in carbonated beverages. Food Chem..

[27] Sun X., Wang K., Gao S., Hong H., Zhang L., Liu H., Feng L., Luo Y. (2021). Purification and characterization of antioxidant peptides from yak (*Bos grunniens*) bone hydrolysates and evaluation of cellular antioxidant activity. J. Food Sci. Technol..

[28] Sarabandi K., Gharehbeglou P., Jafari S.M. (2020). Spray-drying encapsulation of protein hydrolysates and bioactive peptides: Opportunities and challenges. Dry. Technol..

[29] Tonon R.V., Brabet C., Hubinger M.D. (2008). Influence of process conditions on the physicochemical properties of açai (*Euterpe oleraceae* Mart.) powder produced by spray drying. J. Food Eng..

[30] Alghazeer R., Saeed S., Howell N.K. (2008). Aldehyde formation in frozen mackerel (*Scomber scombrus*) in the presence and absence of instant green tea. Food Chem..

[31] Yu M., He S., Tang M., Zhang Z., Zhu Y., Sun H. (2018). Antioxidant activity and sensory characteristics of Maillard reaction products derived from different peptide fractions of soybean meal hydrolysate. Food Chem..

[32] Huang Y., Li H., Huang T., Li F., Sun J. (2014). Lipolysis and lipid oxidation during processing of Chinese traditional smoke-cured bacon. Food Chem..

[33] Alinejad M., Motamedzadegan A., Rezaei M., Regenstein J.M. (2017). The Impact of Drying Method on the Functional and Antioxidant Properties of Whitecheek Shark (*Carcharhinus dussumieri*) Protein Hydrolysates. J. Food Process. Preserv..

[34] Chen H.-M., Muramoto K., Yamauchi F., Nokihara K. (1996). Antioxidant Activity of Designed Peptides Based on the Antioxidative Peptide Isolated from Digests of a Soybean Protein. J. Agric. Food Chem..

[35] Perusko M., Ghnimi S., Simovic A., Stevanovic N., Radomirovic M., Gharsallaoui A., Smiljanic K., Van Haute S., Stanic-Vucinic D., Velickovic T.C. (2021). Maillard reaction products formation and antioxidative power of spray dried camel milk powders increases with the inlet temperature of drying. LWT.

[36] Nalinanon S., Benjakul S., Kishimura H., Shahidi F. (2011). Functionalities and antioxidant properties of protein hydrolysates from the muscle of ornate threadfin bream treated with pepsin from skipjack tuna. Food Chem..

